# Late-adolescent weight categories and early kidney disease in young adulthood: a nationwide study of Arab and Jewish Israelis

**DOI:** 10.1007/s00467-026-07197-7

**Published:** 2026-02-23

**Authors:** Yulia Treister-Goltzman

**Affiliations:** 1 Department of Family Medicine and Siaal Research Center for Family Practice and Primary Care, Faculty of Health Sciences, The Haim Doron Division of Community Health, Ben-Gurion University of the Negev, Beer-Sheva, Israel; 2 Clalit Health Services, Tel Aviv, Israel

**Keywords:** Childhood obesity, Early chronic kidney disease, Cardio-vascular risk factors, Ethnic differences

## Abstract

**Background:**

Previous reports have demonstrated that childhood obesity is associated with early chronic kidney disease. The aim of the study was to assess the relationship between weight categories in late adolescence and early chronic kidney disease (CKD) during young adulthood (age 30 years and younger) in individuals of Jewish and Arab ethnicity in Israel, on a nationwide level.

**Methods:**

This retrospective cohort study included 102,902 adolescents aged 17–19 years – 47,892 of Jewish ethnicity and 53,492 of Arab ethnicity, born between 1988 and 1992 and insured by Clalit Health Services. Early CKD was defined when two urine albumin-to-creatinine ratio tests showed ≥ 30 mg/g within six months of an eGFR test ≥ 60 mL/min/1.73 m^2^.

**Results:**

The incidence (95% CI) of early CKD was higher among adolescents of Arab than of Jewish ethnicity at 52.3 (46.7–58.4) and 34.6 (29.8–39.9) cases per 10^5^ person-years, respectively. The risk for early CKD grew progressively, in adjusted to socio-economic variables models, increasing to HRs (95% CI) of 14.63 (8.86–24.15), and 9.75 (5.35–17.78) in the ‘class 3 obesity’ category among individuals of Arab and Jewish ethnicity, respectively. Sensitivity analyses among participants who had at least one microalbumin-creatinine ratio test and among participants who didn’t develop hypertension and diabetes mellitus during follow-up showed similar patterns.

**Conclusions:**

The findings emphasize the necessity of actions designed to decrease the prevalence of adolescent overweight and obesity, especially in the Arab ethnic minority in Israel. Adolescents with obesity should be monitored closely for signs of early CKD.

**Graphical Abstract:**

**Figure:**
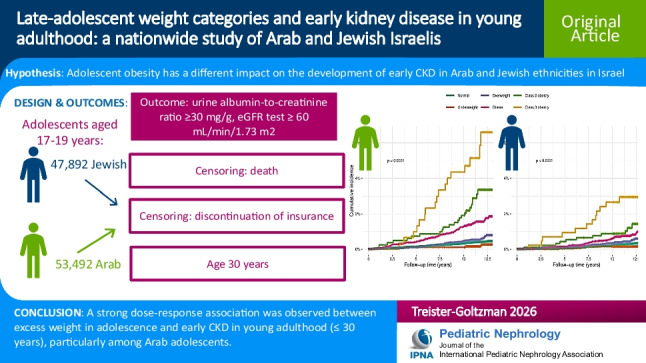
A higher resolution version of the Graphical abstract is available as Supplementary information

**Supplementary Information:**

The online version contains supplementary material available at 10.1007/s00467-026-07197-7.

## Introduction

Adolescent obesity has been linked to adverse health outcomes, including diabetes and cardiovascular diseases [1–6], in adolescence and later life. Kidney damage has been associated with adolescent obesity as well. Proposed mechanisms include metabolic and hemodynamic effects of adiposity, such as insulin resistance, hypertension, and glomerular hyperfiltration. These changes probably remain asymptomatic and unrecognized for a considerable time, culminating in decreased kidney filtration and chronic kidney disease (CKD) [7, 8]. A study on a cohort of 1.2 million adolescents with 25 years of follow-up showed that adolescent obesity increased the risk for advanced CKD from non-diabetic nephropathy more than three-fold [9]. A more recent study showed a dose-dependent association of adolescent obesity with early kidney disease in young adulthood with the hazard ratio reaching 9.4 for those with severe obesity [10].

Microalbuminuria, a marker of early CKD, has been independently associated with increased risks of myocardial infarction, stroke, cardiovascular mortality, and all-cause mortality in large population-based studies [11–14]. Early identification of risk factors for microalbuminuria may therefore offer opportunities for prevention before irreversible kidney damage develops.

The prevalence of obesity among children and adolescents has increased substantially worldwide over recent decades [15]. In Israel, obesity prevalence differs by ethnicity, with consistently higher rates observed among Arab compared with Jewish adolescents [16].

Late adolescence represents a critical period for long-term health, as it coincides with the completion of pubertal development, stabilization of metabolic function, and the establishment of adult behavioral patterns [17]. A recent meta-analysis demonstrated that obesity in late adolescence (after age 17) is a strong predictor of cardiovascular morbidity in adulthood [18]. Israel is characterized by two major ethnic groups, Jewish and Arab, who differ genetically and socio-culturally despite shared regional ancestry [19–21]. Previous studies have reported ethnic differences in obesity-related health outcomes in Israel [22, 23]. Two earlier large studies that assessed the impact of adolescent obesity on CKD during adulthood in Israel were based on pre-army recruitment height and weight measurements, from which the Arab population is exempt.

The goal of this research was to assess the relationship between weight categories in late adolescence and early CKD during young adulthood (age 30 years and younger) in individuals of Jewish and Arab ethnicity in Israel.

## Methods

***Study design and setting*** This was a historical cohort study. The study cohort included adolescents born in 1988–1992 and insured in the “Clalit Health Services” (CHS), the biggest health maintenance organization in Israel which provides services to 52% of the country’s population. Adolescents with documented measurements of BMI at ages 17–19 years were enrolled in the study. Participants with outlier BMI values, major chromosomal abnormalities, other congenital anomalies, and moderate to severe intellectual disabilities, pre-existing albuminuria, overt chronic kidney disease, hypertension, and diabetes mellitus (DM) were excluded. The study period from January 1, 2007 to December 31, 2022, was divided into the exposure period (ages 17–19), when BMI was measured, and the follow-up period. The follow-up period extended from the date of the defining BMI measurement until the earliest of the following: occurrence of an outcome event (first urine albumin-to-creatinine ratio ≥ 30 mg/g), a censoring event (death or discontinuation of insurance in CHS), or reaching 30 years of age. Ages 17–19 years, corresponding to late adolescence, were selected as the exposure period for two main reasons. First, this interval is physiologically and developmentally appropriate for the proposed exposure, because it coincides with the completion of puberty, stabilization of metabolic function, and consolidation of adult behaviors. The distribution of fat becomes fixed with the higher likelihood of visceral adiposity that accelerates cardio-metabolic risk. Second, starting the exposure window in 2007 minimized potential selection bias because, from that year onward, the Israel National Program for Quality Indicators in the Community recommended routine measurement of height and weight for BMI assessment in all adolescents. Since data for this study were collected in 2022, focusing on older adolescents ensured a sufficiently long follow-up period. Due to this technical constraint, early adulthood was defined as ages 20–30 years. The data collected included socio-demographic data, date of death, date of end of insurance period (if relevant), height (cm), weight (kg), BMI, BMI percentile, BMI in early adulthood (ages 20–30), recorded diagnoses of major chromosomal anomalies and intellectual disabilities, kidney pathology, hypertension and DM, the results of urine albumin-creatinine ratio tests and estimated glomerular filtration rate (eGFR). The study was approved by the Ethics Committee of CHS and exempted from the requirement to obtain informed consent (approval #0102–22-COM1).

## Definitions

***Study outcome*** Early CKD was established when 2 urine albumin-creatinine tests showed a ratio of 30 mg/g or greater within six months of an eGFR test of 60 mL/min/1.73 m^2^ or greater. This definition corresponds to stages 1 to 2 CKD, based on the KDIGO (Kidney Disease: Improving Global Outcomes) criteria [24]. According to KDIGO, individuals with G1 (eGFR ≥ 90 mL/min/1.73 m^2^) or G2 (eGFR 60–89 mL/min/1.73 m^2^) kidney function who also show evidence of kidney damage (e.g., albuminuria or structural abnormalities) are classified as having “early CKD,” despite near-normal GFR. This definition has been used in other large-scale studies on the topic [10]. Structural kidney abnormalities were not included in the outcome definition due to the lack of systematic imaging data and their limited relevance to obesity-related early kidney injury. The date of the first positive urine albumin-creatinine test (ratio ≥ 30 mg/g) was considered the date of early CKD onset. Data were collected from both first morning and random urine samples. The eGFR was established based on the Chronic Kidney Disease Epidemiology Collaboration creatinine equation; although it is formally recommended for adults ≥ 18 years, it has been shown to perform well in older adolescents, including those aged 16–18.5 years [25].

BMI was calculated by dividing weight in kilograms by height squared in meters. The main independent variable, ***adolescent weight categories*** was specified according to the U.S. Centers for Disease Control and Prevention (CDC) definition as “underweight” (BMI < 5th percentile), “normal weight” (5th-84.9th percentile), “overweight” (85th-94.9th percentile), “obesity” (≥ 95th percentile, but not including “class 2” and “class 3 obesity”). “Class 2 obesity” was diagnosed if BMI reached ≥ 120% to < 140% of the 95th percentile or BMI ≥ 35 to < 40 kg/m^2^. “Class 3 obesity” was diagnosed if BMI ≥ 140% of the 95th percentile or BMI ≥ 40 kg/m^2^ [26].

***Socioeconomic status (SES)*** in the Clalit computerized database is classified as low, middle, or high based on zip code, with “high SES” representing relatively affluent areas and “low SES” representing relatively deprived areas. CHS covers seven major administrative ***districts*** in Israel. Central districts (Central, Sharon-Shomron, Dan-Petach Tikva, Jerusalem) generally have higher SES and better access to healthcare services, whereas peripheral districts (Northern, Haifa, Southern) often have more limited resources and lower SES [27]. The two main ethnic groups in Israel are Arab and Jewish. In the present study, ***ethnic classification*** was based on the city of residence and the patient’s clinic location. Adolescents who received care in community clinics located in Arab localities, but resided in a predominantly Jewish city, were classified as Arab ethnicity. In Israel, Jewish and Arab populations generally reside in separate towns. In towns with mixed populations, residential neighborhoods are largely segregated by ethnicity, and people typically attend local clinics serving their neighborhood [28, 29]. Therefore, city and clinic location were used as a proxy for ethnicity. This approach is commonly used in large-scale epidemiological and health services research in Israel [30, 31].

Because ICD-10 codes were introduced into the Israeli healthcare system in 2013, while the study period extended from 2007 to 2022, I used ICD-9 codes to ensure uniformity in diagnostic classification when extracting the relevant diagnoses [32]. The diagnosis of ***hypertension*** was established by one of the ICD-9 codes of 401.x- 405.xx, which showed a sensitivity of 65% and a specificity of 90% for the diagnosis of hypertension [33, 34]. ***DM*** was diagnosed by an ICD-9 code of 250.xx, which had a sensitivity of 92% and a specificity of 100% for this diagnosis [35, 36]. In sensitivity analyses, participants who developed these conditions were excluded because they represent strong, independent risk factors for early CKD and could mediate the relationship between obesity and CKD. This approach allowed for the specific assessment of the direct association between adolescent obesity and early CKD. The ***diagnosis of chronic kidney disease including polycystic kidney disease*** was made based on one of the ICD-9 codes, 403.xx, 405. × 1, 582.xx, 583.xx, 585.x, 586, 593.9, which demonstrated a sensitivity of 62–85% and a specificity of 98% [37]. Because major chromosomal and other congenital anomalies, as well as moderate-to-severe intellectual disabilities, may confound the association between BMI and early kidney disease, information on these conditions was collected. These disorders can affect BMI through mechanisms such as altered metabolic rate, abnormal fat distribution, reduced physical activity, medication use, and nutritional challenges. They are also independently associated with kidney disease due to congenital kidney malformations, comorbid systemic disorders, exposure to nephrotoxic medications, and differences in healthcare access. The diagnoses of *major chromosomal and other congenital anomalies* were made in the presence of one of the ICD-9 codes of 758.0–758.3, 758.5–758.9, 759.5–759.9, and *moderate to severe intellectual disabilities* in the presence of ICD-9 codes of 318, and 319. Minor anomalies, isolated structural defects, and mild intellectual disabilities without systemic, metabolic, or developmental impact (e.g., small atrial septal defects or isolated renal hypodysplasia) were not excluded.

### Statistical analyses

I performed data cleaning and identified outlying BMI values. A clinical approach was used to define outliers. Data points that deviated significantly from the rest of the dataset due to obvious errors in data collection or recording were defined as outliers, so-called illegitimate outliers (BMI ≤ 10 and BMI ≥ 60). Adolescents with and without BMI measurements were compared in terms of basic socio-demographic characteristics to assess for possible selection bias. The first BMI measurement of the participants was used for analyses. The baseline characteristics of the study populations were described by ethnic group. Kaplan–Meier survival curves were generated for participants in all weight categories for early CKD in both ethnic groups. Survival curves between the weight groups were compared using the Log-rank test. A log minus log plot and a Schoenfeld residual test were applied to validate the proportionality assumption. Results from the Schoenfeld residual test are presented in Online Resource 1. Cox proportional hazard models were used in each ethnic group to estimate the hazard ratios and 95% confidence intervals (HR (95% CIs)) for early CKD, with the “normal” category being the reference group. HR was then calculated for each ethnic group: unadjusted, adjusted for socio-economic variables (sex, SES) and for socio-economic variables and for adult BMI. A single model was conducted including both populations, with ethnicity included as a covariate to assess whether ethnicity is associated with early kidney disease.

In the sensitivity analyses, crude and adjusted HR (95% CIs) were calculated for participants who had at least one microalbumin-creatinine ratio test during follow-up and for participants who didn’t develop hypertension and diabetes mellitus during follow-up. Two additional sensitivity analyses were performed to assess the potential for selection and information bias. In the first analysis, addressing potential selection bias due to missing BMI values, inverse probability weights were applied to create a pseudo-population that more accurately represents the entire population of insured adolescents. These weights were derived from a logistic regression model predicting the probability of having a BMI measurement based on ethnicity, SES, and sex. In the second analysis, addressing potential information bias from missing urine microalbumin measurements, inverse probability weights were calculated from a logistic regression model predicting the probability of having microalbumin measurements as a function of ethnicity, SES, sex, and weight category. The main analyses were then conducted on these weighted pseudo-populations. I calculated the unadjusted population attributable risk percent (PAR%) for incident early CKD attributable to excessive weight separately for individuals of Arab and Jewish ethnicity as follows:

$$PAR\%=\frac{Pr\left(HR-1\right)}{Pr\left(HR-1\right)+1}\times 100$$

in which Pr is the prevalence of the excessive weight (overweight and all obesity categories together) at the beginning of the follow-up and HR is the unadjusted HR for the early CKD. Adjusted PAR% values were calculated using the same formula, with HRs adjusted for socioeconomic variables, adult BMI, hypertension, and DM. Statistical significance was set at *p* < 0.05.

## Results

**Fig. 1:**
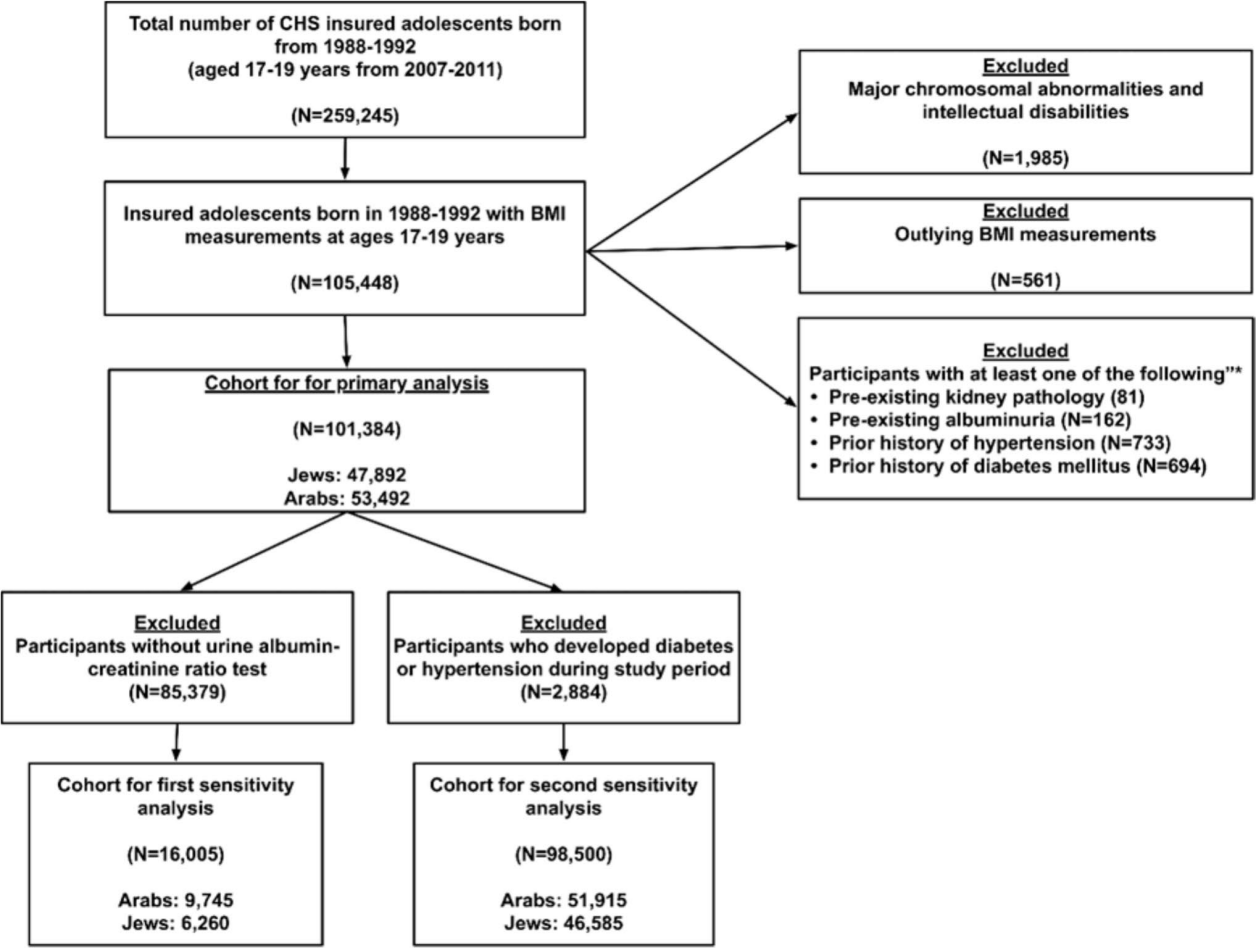
Flowchart of the study cohort selection

Of 259,245 insured adolescents 105,448 had recorded weight and height measurements. Five hundred sixty-one participants with outlying BMI values were excluded. The socio-demographic characteristics of the subsequent cohort were compared to those of adolescents without BMI measurements. Online Resource 2 shows that adolescents without BMI measurements included slightly more males, a higher proportion of individuals of Jewish ethnicity, and more participants from high SES backgrounds. After further exclusion of adolescents with major chromosomal anomalies, moderate to severe intellectual disabilities, adolescents with pre-existing albuminuria, and those diagnosed with kidney disease, hypertension and DM before entry to the study, the final sample consisted of 101,384 participants, 47,892 individuals of Jewish ethnicity and 53,492 individuals of Arab ethnicity (Fig. 1).

The subgroups for sensitivity analyses included 16,005 participants who had at least one microalbumin-creatinine ratio test during follow-up and 98,500 participants who didn’t develop hypertension and diabetes mellitus during follow-up. Table 1 demonstrates the socio-demographic characteristics of the study participants by study group. No differences in sex distribution were observed between individuals of Jewish and Arab ethnicity. Adolescents of Arab ethnicity had a significantly higher BMI compared with their Jewish counterparts. A significantly higher proportion of adolescents of Jewish ethnicity, compared with those of Arab ethnicity, were from higher SES backgrounds and resided in more central districts of Israel.

**Table 1:** Baseline characteristics of the study participants

Variables	Ethnic group		*p-value*
	*Arab* *(53,492)*	*Jewish* *(47,892)*	
Sex (male)	25,282 (47.3)	22,530 (47.0)	0.488
BMI, kg/m^2^			< 0.001
*Median (IQR)*	22.3 (4.8)	21.6 (5.3)	
BMI percentile			< 0.001
*Median (IQR*)	54.5 (57.5)	50.0 (60.8)	
Weight category *N (%):*			< 0.001
*Underweight*	1,900 (3.6)	3,377 (7.1)	
*Normal*	39,083 (73.1)	33,115 (69.1)	
*Overweight*	6,276 (11.7)	4,978 (10.4)	
*Obese*	5,096 (9.5)	5,046 (10.5)	
*Class 2 obesity*	825 (1.5)	944 (2.0)	
*Class 3 obesity*	312 (0.6)	432 (0.9)	
Socio-economic level *N (%):*			< 0.001
*High*	357 (0.7)	8,757 (18.3)	
*Middle*	13,671 (25.6)	33,018 (68.9)	
*Low*	33,253 (62.2)	4,023 (8.4)	
*Missing*	6,211 (11.6)	2,094 (4.4)	
District of residence in Israel			< 0.001
*Central*	947 (1.8)	13,474 (28.1)	
*Northern*	11,196 (20.9)	6,102 (12.7)	
*Haifa*	13,009 (24.3)	7,238 (15.1)	
*Sharon-Shomron*	9,072 (17.0)	5,721 (11.9)	
*Dan-PT*	1,697 (3.2)	5,345 (11.2)	
*Jerusalem*	9,429 (17.6)	3,075 (6.4)	
*Southern*	8,142 (15.2)	6,909 (14.4)	
*Missing*	0 (0.0)	28 (0.1)	

Underweight- BMI < 5th percentile, normal weight- BMI 5th-84.9th percentile, overweight- BMI 85th-94.9th percentile, obesity- BMI ≥ 95th percentile, not including class 2 and class 3 obesity, class 2 obesity- BMI ≥ 120% to < 140% of the 95th percentile or BMI ≥ 35 to < 40 kg/m^2^, class 3 obesity- BMI ≥ 140% of the 95th percentile or BMI ≥ 40 kg/m^2^. IQR- interquartile range

### Incidence of early CKD

**Fig. 2:**
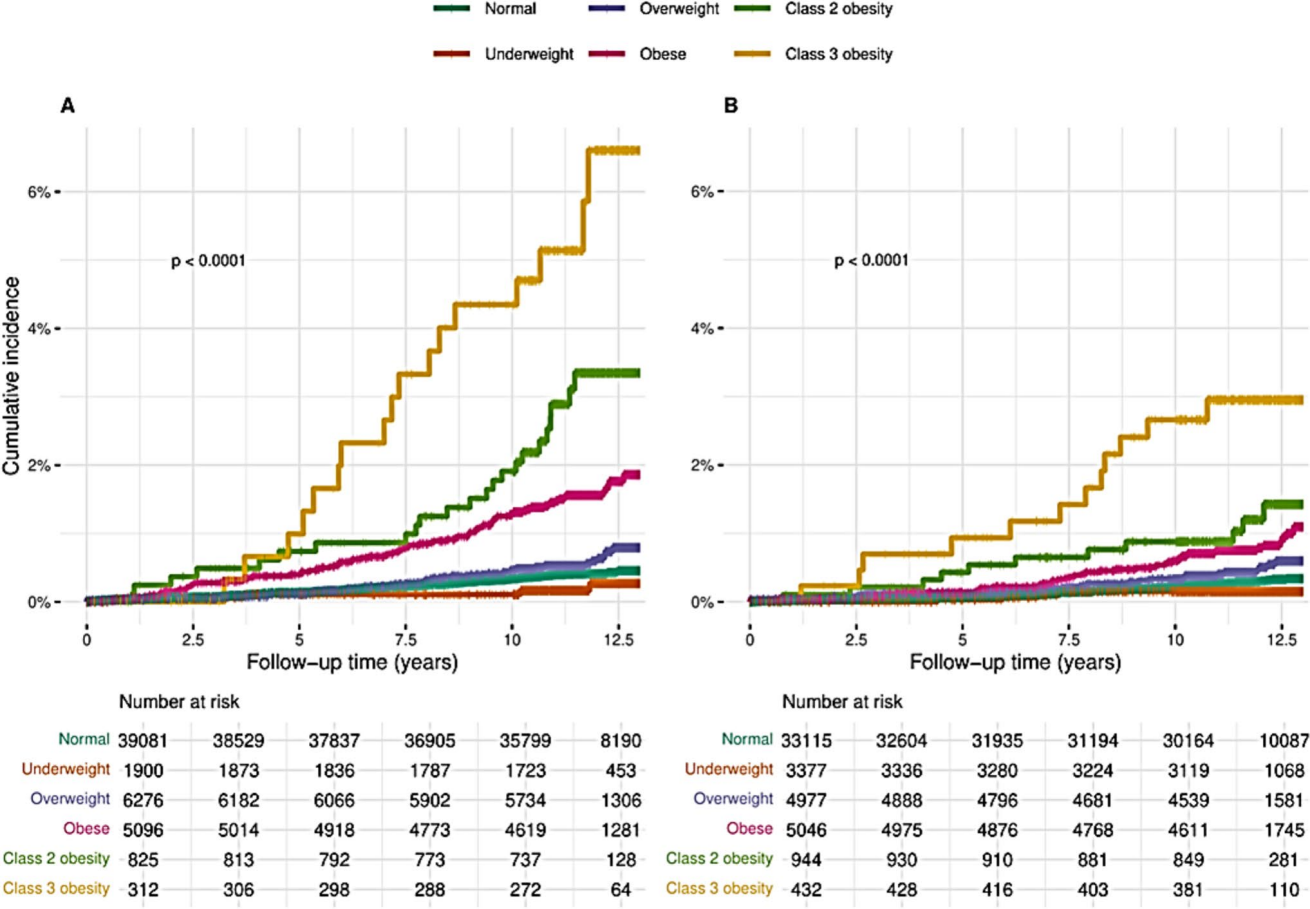
Cumulative incidence of early chronic kidney disease among Arab and Jewish ethnicities by weight group. **A**- Arab ethnicity, **B**- Jewish ethnicity. Underweight- BMI < 5th percentile, normal weight- BMI 5th-84.9th percentile, overweight- BMI 85th-94.9th percentile, obesity- BMI ≥ 95th percentile, not including class 2 and class 3 obesity, class 2 obesity- BMI ≥ 120% to < 140% of the 95th percentile or BMI ≥ 35 to < 40 kg/m^2^, class 3 obesity- BMI ≥ 140% of the 95th percentile or BMI ≥ 40 kg/m.^2^

Table 2 presents the risk estimates for the associations between adolescent weight categories and the incidence of early CKD among adolescents of Arab and Jewish ethnicity. Among adolescents of Arab ethnicity there were 313 incident cases of early CKD in 598,570 person-years of follow-up. The median (interquartile range (IQR)) age at diagnosis was 25.6 (5.1) years. The median (IQR) follow-up for early CKD was 11.7 (1.7) years. The incidence of early CKD demonstrated an incremental increase by BMI group reaching 499.8 diagnoses per 10^5^ person-years in “class 3 obesity” category. The results of the Kaplan–Meier survival analysis (Fig. 2A) and Cox regression model (Table 2) also depict this pattern. Compared with the “normal” reference category, the crude HR (95% CI) for incident early CKD increased across categories to 14.38 (8.71–23.73) in the “class 3” obesity category. When adjusted for sex and socio-economic status, the aHRs (95% CIs) remained stable, while further adjustment for the adult BMI resulted in a more moderate increase in the aHRs (95% CIs) reaching 4.43 (2.47–7.94) in the “class 3” obesity category, compared with the “normal” reference category.

**Table 2:** Risk estimates of the association between adolescent weight categories and incident early kidney disease in young adulthood

	Total	Weight categories in adolescence						P
		Underweight	Normal	Overweight	Obese	Class 2 obesity	Class 3 obesity	
*Arab ethnicity*								
Participants in category, N	53,492	1,900	39,083	6,276	5,096	825	312	
Incident cases, N (%)	313 (0.6)	4 (0.2)	153 (0.4)	37 (0.6)	79 (1.6)	23 (2.8)	17 (5.4)	
Censoring due to death, N (%)	113 (0.2)	4 (0.2)	73 (0.2)	12 (0.2)	21 (0.4)	3 (0.4)	0 (0.0)	
Censoring due to insurance termination, N (%)	5,160 (9.6)	203 (10.7)	3,740 (9.6)	609 (9.7)	496 (9.7)	83 (10.1)	29 (9.3)	
Follow-up, years, Median (IQR)	11.7 (1.7)	11.8 (1.5)	11.7 (1.7)	11.6 (1.5)	11.9 (1.4)	11.4 (1.7)	11.4 (1.9)	< 0.001
Person-years of follow-up	598,569.6	21,420.5	437,183.3	69,836.6	57,667.6	9,060.0	3,401.6	
Incidence (95% CI) (per 10^−5^ person-years)	52.3 (46.7–58.4)	18.7 (5.1- 47.8)	35.0 (29.7- 41.0)	53.0 (37.3- 73.0)	137.0 (108.5–170.7)	253.9 (160.9–380.9)	499.8 (291.1- 800.2)	< 0.001
Age at end of follow-up, years, Median (IQR)	30.0 (0.0)	30.0 (0.0)	30.0 (0.0)	30.0 (0.0)	30.0 (0.0)	30.0 (0.0)	30.0 (0.0)	< 0.001
Age at diagnosis, years, Median (IQR)	25.6 (5.1)	24.4 (7.3)	25.0 (5.8)	26.2 (3.6)	26.3 (5.1)	27.0 (4.5)	25.6 (2.8)	0.368
Adult BMI, kg/m^2^, Median (IQR)	25.4 (6.6)	20.3 (3.8)	24.3 (4.7)	29.3 (5.6)	33.1 (7.0)	40.0 (7.1)	43.8 (10.)	< 0.001
^a^HR (95% CI), *P*-value		0.53 (0.20–1.43) 0.212	Reference	1.52 (1.06–2.17) 0.023	3.89 (2.96–5.10) < 0.001	7.31 (4.71–11.33) < 0.001	14.38 (8.71–23.73) < 0.001	
^b^aHR (95% CI) *P*-value		0.53 (0.20–1.45) 0.221		1.51 (1.05–2.16) 0.025	3.95 (3.01–5.18) < 0.001	7.37 (4.76–11.43) < 0.001	14.63 (8.86–24.15) < 0.001	
^c^aHR (95% CI) *P*-value		0.70 (0.26- 1.88) 0.476		1.12 (0.78–1.61) 0.548	2.24 (1.65–3.03) < 0.001	2.94 (1.80–4.80) < 0.001	4.43 (2.47–7.94) < 0.001	
*Jewish ethnicity*								
Participants in category, N	47,892	3,377	33,115	4,978	5,046	944	432	
Incident cases, N (%)	190 (0.4)	5 (0.1)	96 (0.3)	24 (0.5)	42 (0.8)	11 (1.2)	12 (2.8)	
Censoring due to death, N (%)	85 (0.2)	8 (0.2)	57 (0.2)	8 (0.2)	11 (0.2)	1 (0.1)	0 (0.0)	
Censoring due to insurance termination, N (%)	4,965 (10.4)	306 (9.1)	3,484 (10.5)	525 (10.5)	496 (9.8)	108 (11.4)	46 (10.6)	
Follow-up, years, Median (IQR)	12.1 (1.4)	12.1 (1.2)	12.1 (1.4)	12.1 (1.5)	12.2 (1.2)	11.9 (1.5)	11.8 (1.7)	< 0.001
Person-years of follow-up	549,436.8	39,257.0	379,172.0	56,936.7	58,520.1	10,715.9	4,835.1	
Incidence (95% CI) (per 10^−5^ person-years)	34.6 (29.8–39.9)	12.7 (4.1–29.7)	25.3 (20.5–30.9)	42.2 (27.0–62.7)	71.8 (51.7–97.0)	102.7 (51.2–183.7)	248.2 (128.2–433.5)	< 0.001
Age at end of follow-up, years, Median (IQR)	30.0 (0.0)	30.0 (0.0)	30.0 (0.0)	30.0 (0.0)	30.0 (0.0)	30.0 (0.0)	30.0 (0.0)	0.018
Age at diagnosis, years, Median (IQR)	26.2 (5.6)	24.4 (1.3)	26.0 (5.7)	26.4 (4.3)	26.9 (4.9)	25.3 (6.0)	25.3 (4.4)	0.462
Adult BMI, kg/m^2^, Median (IQR)	24.5 (6.9)	19.7 (3.4)	23.5 (4.6)	28.9 (5.9)	33.2 (7.8)	40.3 (7.3)	44.4 (9.9)	< 0.001
^a^HR (95% CI), *P*-value		0.50 (0.20–1.23) 0.131	Reference	1.66 (1.06–2.60) 0.026	2.81 (1.96–4.04) < 0.001	4.08 (2.18–7.61) < 0.001	9.96 (5.47–18.16) < 0.001	
^b^aHR (95% CI) *P*-value		0.50 (0.21–1.24) 0.136		1.65 (1.06–2.59) 0.027	2.81 (1.96–4.06) < 0.001	3.99 (2.13–7.45) < 0.001	9.75 (5.35–17.78) < 0.001	
^c^aHR (95% CI) *P*-value		0.64 (0.26- 1.59) 0.341		1.16 (0.73–1.83) 0.541	1.47 (0.96–2.25) 0.075	1.46 (0.72–2.95) 0.296	2.60 (1.23–5.49) 0.012	

Underweight- BMI < 5th percentile, normal weight- BMI 5th-84.9th percentile, overweight- BMI 85th-94.9th percentile, obese- BMI ≥ 95th percentile, not including class 2 and class 3 obesity, class 2 obesity- BMI ≥ 120% to < 140% of the 95th percentile or BMI ≥ 35 to < 40 kg/m^2^, class 3 obesity- BMI ≥ 140% of the 95th percentile or BMI ≥ 40 kg/m^2^. IQR- interquartile range*,* SD- standard deviation, 95% CI- 95% of the confidence interval, HR- Hazard ratio, aHR- adjusted Hazard ratio

^a^Unadjusted, ^b^Adjusted to socio-economic factors, ^c^ Adjusted to socio-economic factors and adult BMI

One hundred and ninety Jewish adolescents developed early CKD during 549,437 person-years of follow-up. The incidence of diagnosed early CKD was lower than in the Arab ethnic group in all weight categories and rose steadily reaching 248.2 per 10^5^ person-years in “class 3” obesity category. The median (IQR) of the follow-up and age at diagnosis were similar to those in the Arab group at 12.1 (1.4) and 26.2 (5.6), respectively. The Kaplan–Meier analysis (Fig. 2B) displayed a separation of the curve of the “class 3” obesity after the first year of follow-up, and other weight categories after around 5 years of follow-up. The crude and adjusted for socio-demographic variables and adult BMI HRs (95% CIs) for early CKD increased across obesity categories, reaching 9.96 (5.47–18.16) and 2.60 (1.23–5.49), respectively, in the “class 3” obesity category, compared with the reference category (Table 2).

The lower risk of early CKD among individuals of Jewish compared with Arab ethnicity was apparent in the single model applied to the entire study cohort, with HRs (95% CIs) of 0.63 (0.52–0.75) in the unadjusted model and 0.76 (0.60–0.95) in the fully adjusted model (Online Resource 3). Online Resource 4 presents a fully adjusted model including the interaction between ethnicity and BMI. This analysis showed that the association between BMI and early CKD differed by ethnicity, with a smaller magnitude among Jewish compared with Arab adolescents (aHR 0.97, 95% CI 0.95–0.99).

### Sensitivity analyses

Table 3 presents the results of the main sensitivity analyses. In the Arab ethnic group, an incremental increase in odds for early CKD with increase in weight category was observed in both sensitivity analyses. The adjusted for socio-economic variables HRs (95% CI) for early CKD reached 5.83 (3.53–9.62) in the “class 3” obesity category compared with the “normal” weight category for participants, who had at least one microalbumin-creatinine ratio test during follow-up and 9.01 (3.95–20.54) for participants who didn’t develop hypertension and DM during follow-up. This increase was more moderate than in the main analysis and began from the “obese” weight category with no association seen in the “overweight” category.

**Table 3:** Sensitivity analyses on the association between adolescent weight categories and incident early kidney disease in young adulthood

	Total	Weight categories in adolescence						P
		Underweight	Normal	Overweight	Obese	Class 2 obesity	Class 3 obesity	
*Arab ethnicity*								
*Sensitivity analysis (1)- risk for early CKD among participants with at least one microalbumin-creatinine ratio test during follow-up*								
^a^HR (95% CI), *P*-value		0.71 (0.26–1.91) 0.493	Reference	1.08 (0.75–1.54) 0.693	2.21 (1.69–2.91) < 0.001	2.98 (1.92–4.62) < 0.001	5.79 (3.51–9.56) < 0.001	
^b^aHR (95% CI) *P*-value		0.71 (0.26–1.92) 0.502		1.07 (0.75–1.53) 0.716	2.21 (1.68–2.90) < 0.001	3.02 (1.95–4.69) < 0.001	5.83 (3.53–9.62) < 0.001	
^c^aHR (95% CI) *P*-value		0.84 (0.31- 2.27) 0.728		0.90 (0.62–1.31) 0.583	1.60 (1.16–2.21) 0.004	1.87 (1.12–3.13) 0.017	3.10 (1.68–5.72) < 0.001	
*Sensitivity analysis (2)- risk for early CKD among participants who didn’t develop hypertension and diabetes mellitus during follow-up*								
^a^HR (95% CI), *P*-value		0.40 (0.10–1.63) 0.201	Reference	1.08 (0.65–1.81) 0.767	2.15 (1.41–3.29) < 0.001	3.12 (1.37–7.11) 0.007	8.77 (3.85–19.99) < 0.001	
^b^aHR (95% CI) *P*-value		0.42 (0.10–1.68) 0.219		1.08 (0.65–1.81) 0.767	2.21 (1.45–3.39) < 0.001	3.17 (1.39–7.21) 0.006	9.01 (3.95–20.54) < 0.001	
^c^aHR (95% CI) *P*-value		0.48 (0.12–1.97) 0.311		0.90 (0.53–1.54) 0.711	1.55 (0.95–2.52) 0.081	1.85 (0.75–4.58) 0.183	4.58 (1.76–11.92) 0.002	
*Jewish ethnicity*								
*Sensitivity analysis (1)- risk for early CKD among participants with at least one microalbumin-creatinine ratio test during follow-up*								
^a^HR (95% CI), *P*-value		0.49 (0.20–1.20) 0.117	Reference	1.34 (0.86–2.09) 0.202	1.67 (1.16–2.40) 0.005	1.83 (0.98–3.41) 0.059	3.73 (2.05–6.80) < 0.001	
^b^aHR (95% CI) *P*-value		0.49 (0.20–1.20) 0.116		1.33 (0.85–2.09) 0.207	1.67 (1.16–2.40) 0.006	3.81 (0.97–3.39) 0.062	3.72 (2.04–6.78) < 0.001	
^c^aHR (95% CI) *P*-value		0.53 (0.21–1.30) 0.165		1.18 (0.74–1.90) 0.489	1.36 (0.86–2.14) 0.191	1.33 (0.63–2.81) 0.449	2.46 (1.09–5.57) 0.031	
*Sensitivity analysis (2)- risk for early CKD among participants who didn’t develop hypertension and diabetes mellitus during follow-up*								
^a^HR (95% CI), *P*-value		0.38 (0.12–1.22) 0.103	Reference	0.73 (0.35–1.50) 0.387	1.72 (1.04–2.85) 0.034	1.56 (0.49–4.95) 0.450	4.74 (1.73–12.97) 0.002	
^b^aHR (95% CI) *P*-value		0.38 (0.12–1.22) 0.107		0.72 (0.35–1.50) 0.386	1.74 (1.05–2.87) 0.032	1.53 (0.48–4.86) 0.470	4.66 (1.70–12.76) 0.003	
^c^aHR (95% CI) *P*-value		0.46 (0.14–1.47) 0.190		0.56 (0.27–1.19) 0.132	1.10 (0.61–2.00) 0.755	0.75 (0.21–2.62) 0.650	1.85 (0.56–6.13) 0.312	

Underweight- BMI < 5th percentile, normal weight- BMI 5th-84.9th percentile, overweight- BMI 85th-94.9th percentile, obese- BMI ≥ 95th percentile, not including class 2 and class 3 obesity, class 2 obesity- BMI ≥ 120% to < 140% of the 95th percentile or BMI ≥ 35 to < 40 kg/m^2^, class 3 obesity- BMI ≥ 140% of the 95th percentile or BMI ≥ 40 kg/m^2^. SD- standard deviation, 95% CI- 95% of the confidence interval, HR- Hazard ratio, aHR- adjusted Hazard ratio

^a^Unadjusted, ^b^Adjusted to socio-economic factors, ^c^ Adjusted to socio-economic factors and adult BMI

For adolescents of Jewish ethnicity, a similar pattern of a more moderate increase throughout the weight categories, beginning from the “obese” category, was seen. The HRs (95% CIs) for early CKD in the “class 3” obesity category, adjusted for socio-economic variables, were 3.72 (2.04–6.78) among participants who had at least one microalbumin-creatinine ratio test, and 4.66 (1.70–12.76) among participants who did not develop hypertension or DM during follow-up, compared with the reference weight category. Notably, HRs did not reach statistical significance in the “class 2” category in the Jewish group.

In the sensitivity analyses, HRs adjusted for adult BMI showed no association in most excessive weight categories for either ethnic group.

Additional sensitivity analyses using inverse probability weighting for missing BMI values and missing microalbumin values are presented in Online Resources 5 and 6, respectively. The first analysis yielded results that were nearly identical to those of the main analysis. In the second analysis, a more moderate increase across weight categories, beginning with the “obese” category, was observed compared with the main analysis. In this analysis no association was observed in the “overweight” category in either ethnic group or in the “class 2” category within the Jewish group.

### Population attributable risk

Based on the observed prevalence of 23.4% and 23.8% for BMI < 85th percentile during the exposure period among adolescents of Arab and Jewish ethnicity, respectively, the crude fractions of early CKD attributable to excess weight were 33.4% for adolescents of Arab ethnicity and 28.7% for adolescents of Jewish ethnicity. After adjustment for sociodemographic variables, adult BMI, hypertension, and DM, the attributable fractions were 7.8% for individuals of Arab ethnicity and 3.0% for individuals of Jewish ethnicity.

## Discussion

The present study found a significant association between adolescent weight categories and early CKD in the two major Israeli ethnic groups, Arab and Jewish. This association was pronounced in Arab and Jewish adolescents, reaching, after adjustment for socio-economic variables, HRs of 14.4 and 9.8 in the “class 3” obesity category, respectively. This association persisted among adolescents without diabetes and hypertension throughout follow-up, albeit with a lower strength of association. Compared with adolescents of Jewish ethnicity, Arab adolescents had an increased risk of early CKD. This study supports the adverse cardiometabolic consequences of obesity in late adolescence; however, no comparisons with younger age groups could be made. There is a consensus on the association of adult BMI with kidney disease, both early kidney dysfunction and advanced kidney failure, even in metabolically normal obesity [8, 38, 39]. The association of adolescent obesity with kidney disease is an emerging topic, with a few recent large-scale cohort studies showing this association [9, 10, 40, 41]. These studies showed evidence for advanced kidney disease and for kidney-related mortality later in life [9, 37], for early kidney disease in young adulthood [10], and even for early kidney damage already in adolescence [41].

A growing body of evidence indicates that there are ethnic disparities in early and advanced kidney disease, regardless of the presence of cardio-metabolic diseases [42–44]. The pathogenetic mechanisms for these disparities are not clear, so the studies highlighted the need for further research to clarify the specific pathways causing CKD across diverse racial/ethnic groups.

The results of the present study revealed that adolescent BMI has a different impact on the development of early CKD among individuals of Arab and Jewish ethnicity. There are several possible explanations for this finding. The first is different genetic and environmental components of BMI in the two populations. A Mendelian randomization study supported a genetic basis for such differences in overall and cause-specific CKD [45]. There are reports that genetically high rather than environmentally mediated BMI, has a much stronger influence on CKD development [46]. Another possible explanation is different typical body fat locations (such as abdominal obesity) in different ethnic groups. Abdominal obesity in young adults was independently associated with albuminuria even after controlling for blood pressure and glucose levels [47]. Another large study provided evidence that in some ethnic groups visceral adiposity index outperforms BMI and waist circumference in effectively predicting the risk of CKD [48]. The stronger association between adolescent obesity and early CKD observed among individuals of Arab ethnicity may reflect differences in socioeconomic and structural determinants of health rather than intrinsic ethnic susceptibility. Arab communities in Israel often experience lower socioeconomic status, including reduced income, education, and healthcare access, which are established risk factors for both obesity and CKD [21]. These social and environmental disadvantages can amplify the impact of obesity on kidney health, suggesting that the observed disparity is largely driven by modifiable social determinants rather than ethnicity per se [49]. Participants in whom incident CKD could be attributed to causes other than BMI were excluded in this study by design. This was implemented by excluding participants with pre-existing DM and hypertension and ensuring that the initial presentation was not accompanied by a pronounced reduction in eGFR. Further exclusion of the participants who developed DM and hypertension during follow-up preserved the dose-dependent association between weight categories and incident CKD and ethnic differences in the strength of this association. The reduced intensity of the association in this sensitivity analysis probably stems from the fact that adolescent obesity plays a role also in the development of DM and hypertension, so exclusion of cases with these diseases could lead to the exclusion of some of the incident early CKD cases. Adjustment for adult BMI resulted in decreased strength and even disappearance of statistical significance in some of the associations, suggesting at least a partial mediating role for adult obesity in this association.

The findings of the present study have important implications for Israeli healthcare organizations, highlighting the need for early, targeted prevention strategies aimed at adolescents with obesity. The markedly elevated risk of early CKD, particularly among adolescents with severe obesity, suggests that intensified screening, weight-management interventions, and long-term kidney monitoring may be warranted within community and school-based health services. The higher risk observed among Arab adolescents further underscores the importance of culturally tailored prevention programs and equitable allocation of healthcare resources across ethnic groups. From a healthcare system perspective, early identification and intervention during adolescence may reduce the future burden of CKD, associated cardiovascular morbidity, and healthcare costs borne by Israeli health maintenance organizations.

### Limitations and strengths

The primary limitation of this study is that the cohort included only adolescents with recorded BMI measurements, which could introduce selection bias. However, the universal recommendation to measure BMI in all adolescents aged 14–19 years, combined with the similar results obtained from analyses using the inverse probability weighting method, minimizes the likelihood of such bias. Residual selection bias due to unmeasured or unobserved factors cannot be excluded. The second major limitation is that not all adolescents underwent microalbumin testing. However, the results were not significantly different in the sensitivity analysis limited to adolescents who underwent testing. This study did not capture individuals with preserved eGFR and isolated structural kidney abnormalities without albuminuria, which may have led to under-ascertainment of early CKD. Another limitation is the inability to adjust for variables that may influence the development of early CKD but cannot be captured in a study based on medical records, such as diet and physical activity, or were not available for the present study, including prematurity, autoimmune diseases, and acute kidney injury. The low sensitivity of the diagnoses excluded in the sensitivity analyses (hypertension, DM, and chronic kidney disease) may have resulted in under-identification of these conditions and, consequently, residual confounding. Ethnicity was defined based on city and clinic of residence rather than individual self-report, which may result in some misclassification. However, given the high degree of residential segregation between Jewish and Arab populations in Israel, this proxy likely reflects the predominant ethnic affiliation of the participants. Additionally, the study was conducted by a single investigator; while all analyses were pre-specified, carefully documented, and performed using validated methods within a secure environment, independent team verification was not feasible.

Lastly, BMI has limitations as a useful measure of obesity. Its utility differs in different ethnic groups, and it cannot distinguish between muscle and fat tissue [49]. At the same time, BMI is still the most widely used measure in population-based epidemiological studies and has a good correlation with body fat percentage [50].

The primary strength of this study is using a single large, reliable and nationally representative database that contained exposure variables, outcome variables and potential confounders without a need for linkage to other external sources. An additional strength of the present study is that the BMI measurements and the recording of the diagnoses were carried out by medical staff. Dividing into weight categories, instead of simply referring to “obese” and “nonobese” groups, enabled identification of subtle distinctions in the associations across the categories.

### Conclusions and implications

Adolescent obesity was strongly associated with the development of early CKD in both Jewish and Arab populations. Notably, increasing BMI was associated with a disproportionately higher risk of early CKD among Arab adolescents compared with Jewish adolescents. These results suggest that while obesity prevention is essential in all populations, Arab adolescents may be particularly vulnerable to the adverse kidney effects of excess body weight. Adolescents with excessive weight should be monitored closely for signs of early CKD, which is strongly associated with mortality.

## Supplementary Information

Below is the link to the electronic supplementary material.

Graphical abstract 297 KB)

Supplementary file2 (DOCX 25 KB)

Supplementary file3 (DOCX 24 KB)

Supplementary file4 (DOCX 24 KB)

Supplementary file5 (DOCX 23 KB)

Supplementary file6 (DOCX 26 KB)

Supplementary file7 (DOCX 26 KB)

## Data Availability

The data that support the findings of this study are available from Clalit Health Services. Restrictions apply to the availability of these data, which were used under license for this study.
