# Supplementary material for: Late-adolescent weight categories and early kidney disease in young adulthood: a nationwide study of Arab and Jewish Israelis
Source: Pediatr Nephrol. 2026 Feb 23;41(7):2131–43. doi: 10.1007/s00467-026-07197-7 (PMC13197338; doi:10.1007/s00467-026-07197-7)
Supplement: Supplementary file 1 — Graphical abstract 297 KB) [file 467_2026_7197_MOESM1_ESM.pptx]

## Slide 1
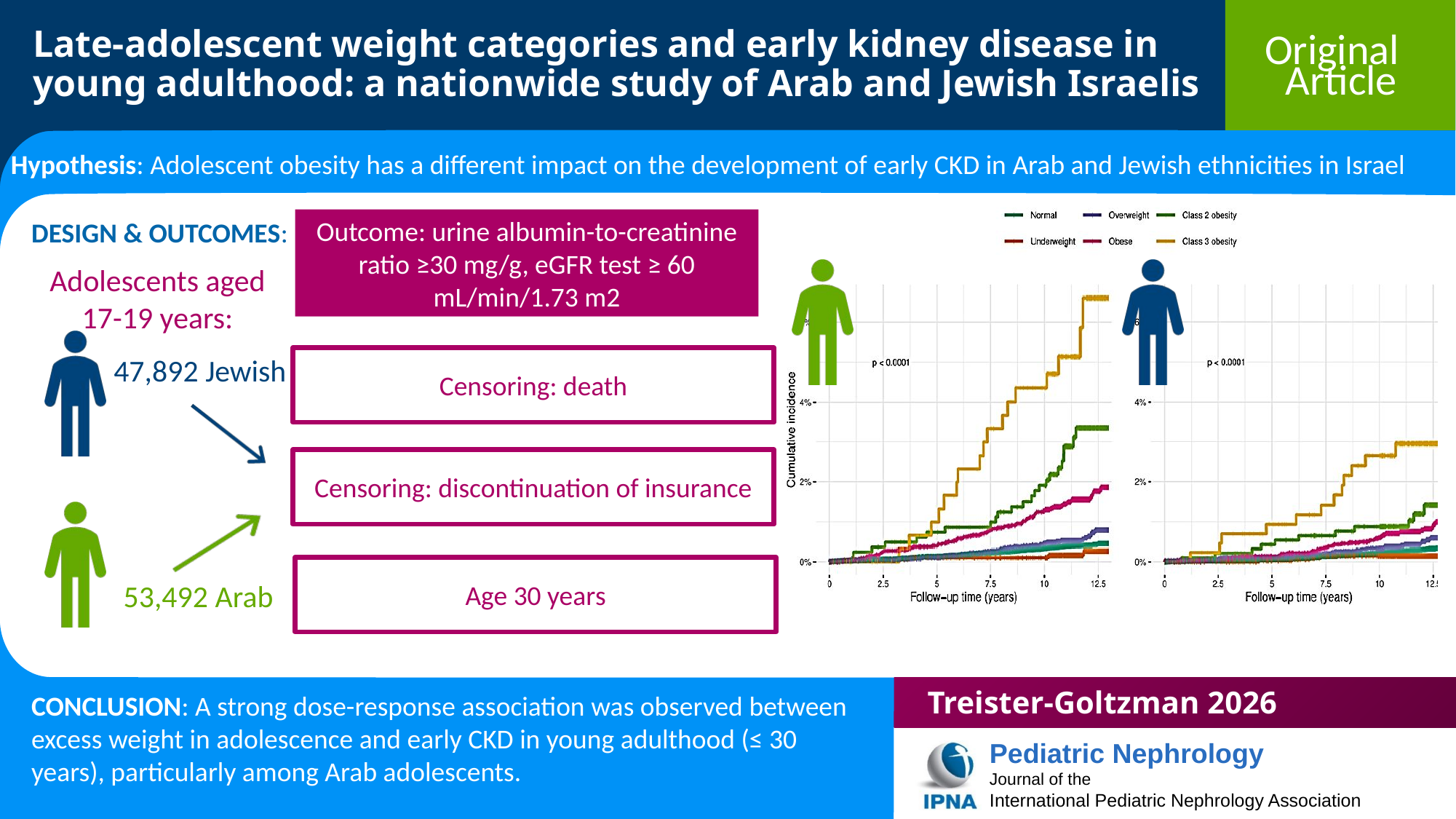

Late-adolescent weight categories and early kidney disease in young adulthood: a nationwide study of Arab and Jewish Israelis
Hypothesis: Adolescent obesity has a different impact on the development of early CKD in Arab and Jewish ethnicities in Israel
Outcome: urine albumin-to-creatinine ratio ≥30 mg/g, eGFR test ≥ 60 mL/min/1.73 m2
DESIGN & OUTCOMES:
Adolescents aged 17-19 years:
47,892 Jewish
Censoring: death
Censoring: discontinuation of insurance
Age 30 years
53,492 Arab
Treister-Goltzman 2026
CONCLUSION: A strong dose-response association was observed between excess weight in adolescence and early CKD in young adulthood (≤ 30 years), particularly among Arab adolescents.
