# Supplementary material for: Late-adolescent weight categories and early kidney disease in young adulthood: a nationwide study of Arab and Jewish Israelis
Source: Pediatr Nephrol. 2026 Feb 23;41(7):2131–43. doi: 10.1007/s00467-026-07197-7 (PMC13197338; doi:10.1007/s00467-026-07197-7)
Supplement: Supplementary file 2 — Supplementary file2 (DOCX 25 KB) [file 467_2026_7197_MOESM2_ESM.docx]

**Article title:** Ethnic differences in the association of weight categories in adolescence with early kidney disease in young adulthood - a nationwide study

**Journal name:** Pediatric Nephrology

**Author names:** Yulia Treister-Goltzman

**Affiliation and e-mail address of the corresponding author:** Yulia Treister-Goltzman, [yuliatr@walla.com](mailto:yuliatr@walla.com)

**Online Resource 1.** Results of the Schoenfeld residual test

| **Variable** | **Chi-square statistic** | **Degrees of freedom** | **p-value** |
| --- | --- | --- | --- |
| *Whole population* | | | |
| Ethnicity | 0.91 | 1 | 0.339 |
| Weight category | 5.43 | 5 | 0.365 |
| Sex | 3.50 | 1 | 0.062 |
| Socio-economics status | 4.40 | 2 | 0.111 |
| Global | 14.25 | 9 | 0.114 |
| *Arab ethnicity* | | | |
| Weight category | 7.36 | 5 | 0.195 |
| Sex | 3.70 | 1 | 0.054 |
| Socio-economics status | 3.80 | 2 | 0.164 |
| Global | 14.86 | 8 | 0.065 |
| *Jewish ethnicity* |  |  |  |
| Weight category | 3.25 | 5 | 0.661 |
| Sex | 0.23 | 1 | 0.634 |
| Socio-economics status | 2.04 | 2 | 0.359 |
| Global | 4.71 | 8 | 0.077 |
|  |  |  |  |
