# Supplementary material for: Late-adolescent weight categories and early kidney disease in young adulthood: a nationwide study of Arab and Jewish Israelis
Source: Pediatr Nephrol. 2026 Feb 23;41(7):2131–43. doi: 10.1007/s00467-026-07197-7 (PMC13197338; doi:10.1007/s00467-026-07197-7)
Supplement: Supplementary file 3 — Supplementary file3 (DOCX 24 KB) [file 467_2026_7197_MOESM3_ESM.docx]

**Article title:** Ethnic differences in the association of weight categories in adolescence with early kidney disease in young adulthood - a nationwide study

**Journal name:** Pediatric Nephrology

**Author names:** Yulia Treister-Goltzman

**Affiliation and e-mail address of the corresponding author:** Yulia Treister-Goltzman, [yuliatr@walla.com](mailto:yuliatr@walla.com)

**Online Resource 2.** Comparison of adolescents with and without BMI measurements

| **Variable** | **Adolescents, with measurements**  **(N=104,887)** | **Adolescents without measurements**  **(N=154,359)** |
| --- | --- | --- |
| **Sex (male),**  *N (%)* | 49,623 (47.3) | 79,795 (51.7) |
| **Ethnicity (Jewish),**  *N (%)* | 54,929 (52.4) | 82,509 (53.5) |
| **Socio-economic status,** N (%):  *low*  *medium*  *high*  *no data* | 38,317 (36.5)  48,490 (46.2)  9,501 (9.1)  8,579 (8.2) | 48,529 (31.4)  75,774 (49.1)  18,138 (11.8)  11,918 (7.7) |
| **District of residency,** N (%):  *Northern*  *Haifa*  *Sharon-Shomron*  *Central*  *Dan-Petah-Tikva*  *Jerusalem*  *Southern*  *No data* | 17,820 (17.0)  20,961 (20.0)  15,256 (14.5)  15,014 (14.3)  7,313 (7.0)  12,864 (12.3)  15,630 (14.9)  29 (0.0) | 25,842 (16.7)  29,856 (19.3)  21,086 (13.7)  21,744 (14.1)  10,929 (7.1)  22,875 (14.8)  21,980 (14.2)  47 (0.0) |
