# Supplementary material for: Late-adolescent weight categories and early kidney disease in young adulthood: a nationwide study of Arab and Jewish Israelis
Source: Pediatr Nephrol. 2026 Feb 23;41(7):2131–43. doi: 10.1007/s00467-026-07197-7 (PMC13197338; doi:10.1007/s00467-026-07197-7)
Supplement: Supplementary file 4 — Supplementary file4 (DOCX 24 KB) [file 467_2026_7197_MOESM4_ESM.docx]

**Article title:** Ethnic differences in the association of weight categories in adolescence with early kidney disease in young adulthood - a nationwide study

**Journal name:** Pediatric Nephrology

**Author names:** Yulia Treister-Goltzman

**Affiliation and e-mail address of the corresponding author:** Yulia Treister-Goltzman, [yuliatr@walla.com](mailto:yuliatr@walla.com)

**Online Resource 3.** Association between adolescent weight categories and incident early kidney disease in young adulthood in the whole population of participants (N=101,384)

|  | | ^a^HR (95% CI),  P-value | ^b^aHR (95% CI)  P-value | ^c^aHR (95% CI)  P-value |
| --- | --- | --- | --- | --- |
| Weight category in adolescence | Underweight | 0.52 (0.27-1.02)  0.059 | 0.53 (0.27-1.04)  0.063 | 0.68 (0.35-1.33)  0.265 |
|  | Normal (reference) |  | | |
|  | Overweight | 1.57 (1.19-2.08)  0.002 | 1.56 (1.18-2.07)  0.002 | 1.14 (0.85-1.51)  0.377 |
|  | Obese | 3.45 (2.78-4.29)  <0.001 | 3.49 (2.81-4.34)  <0.001 | 1.93 (1.51-2.47)  <0.001 |
|  | Class 2 obesity | 5.87 (4.10-8.41)  <0.001 | 5.85 (4.09-8.38)  <0.001 | 2.28 (1.52-3.4)  <0.001 |
|  | Class 3 obesity | 12.32 (8.38-18.11)  <0.001 | 12.32 (8.38-18.11)  <0.001 | 3.60 (2.27-5.69)  <0.001 |
| Ethnicity (Jewish) | | 0.63 (0.52- 0.75)  <0.001 | 0.74 (0.59-0.93)  0.009 | 0.76 (0.60-0.95)  0.015 |

HR- Hazard ratio, aHR- adjusted Hazard ratio

^a^Unadjusted, ^b^Adjusted to socio-economic factors, ^c^ Adjusted to socio-economic factors and adult BMI.

Underweight- BMI <5th percentile, normal weight- BMI 5th-84.9th percentile, overweight- BMI 85th-94.9th percentile, obesity- BMI ≥95th percentile, not including class 2 and class 3 obesity, class 2 obesity- BMI ≥120% to <140% of the 95^th^ percentile or BMI ≥35 to <40 kg/m^2^, class 3 obesity- BMI ≥140% of the 95^th^ percentile or BMI ≥40 kg/m^2^
