# Supplementary material for: Late-adolescent weight categories and early kidney disease in young adulthood: a nationwide study of Arab and Jewish Israelis
Source: Pediatr Nephrol. 2026 Feb 23;41(7):2131–43. doi: 10.1007/s00467-026-07197-7 (PMC13197338; doi:10.1007/s00467-026-07197-7)
Supplement: Supplementary file 5 — Supplementary file5 (DOCX 23 KB) [file 467_2026_7197_MOESM5_ESM.docx]

**Article title:** Ethnic differences in the association of weight categories in adolescence with early kidney disease in young adulthood - a nationwide study

**Journal name:** Pediatric Nephrology

**Author names:** Yulia Treister-Goltzman

**Affiliation and e-mail address of the corresponding author:** Yulia Treister-Goltzman, [yuliatr@walla.com](mailto:yuliatr@walla.com)

**Online Resource 4.** Association between adolescent BMI and incident early kidney disease in young adulthood: interaction term analysis

|  | aHR (95% CI)  P-value |
| --- | --- |
| BMI in adolescence | 1.07 (1.05-1.09)  <0.001 |
| BMI*Ethnicity (Jewish) | 0.97 (0.95-0.99)  0.030 |

aHR- adjusted Hazard ratio (to socio-economic factors and adult BMI).
