# Supplementary material for: Late-adolescent weight categories and early kidney disease in young adulthood: a nationwide study of Arab and Jewish Israelis
Source: Pediatr Nephrol. 2026 Feb 23;41(7):2131–43. doi: 10.1007/s00467-026-07197-7 (PMC13197338; doi:10.1007/s00467-026-07197-7)
Supplement: Supplementary file 6 — Supplementary file6 (DOCX 26 KB) [file 467_2026_7197_MOESM6_ESM.docx]

**Article title:** Ethnic differences in the association of weight categories in adolescence with early kidney disease in young adulthood - a nationwide study

**Journal name:** Pediatric Nephrology

**Author names:** Yulia Treister-Goltzman

**Affiliation and e-mail address of the corresponding author:** Yulia Treister-Goltzman, [yuliatr@walla.com](mailto:yuliatr@walla.com)

**Online Resource 5.** Association between adolescent weight category and incident early kidney disease in young adulthood using inverse probability weighting for missing BMI values

|  | **Total** | **Weight categories in adolescence** | | | | | |
| --- | --- | --- | --- | --- | --- | --- | --- |
|  |  | **Underweight** | **Normal** | **Overweight** | **Obese** | **Class 2 obesity** | **Class 3 obesity** |
| ***Arab ethnicity*** | | | | | | | |
| Participants in category, N | 53,492 | 1,900 | 39,083 | 6,276 | 5,096 | 825 | 312 |
| ^a^HR (95% CI),  P-value |  | 0.52 (0.19-1.39)  0.192 | Reference | 1.54 (1.07-2.20)  0.019 | 3.96 (3.02-5.20)  <0.001 | 7.41 (4.78-11.50)  <0.001 | 14.75 (8.94-24.35)  <0.001 |
| ^b^aHR (95% CI)  P-value |  | 0.52 (0.20-1.45)  0.200 |  | 1.53 (1.07-2.20)  0.020 | 4.02 (3.07-5.27)  <0.001 | 7.48 (4.83-11.60)  <0.001 | 14.99 (9.10-24.69)  <0.001 |
| ^c^aHR (95% CI)  P-value |  | 0.68 (0.25- 1.82)  0.439 |  | 1.14 (0.79-1.63)  0.489 | 2.28 (1.72-3.03)  <0.001 | 2.98 (1.87-4.76)  <0.001 | 4.55 (2.68-7.73)  <0.001 |
| ***Jewish ethnicity*** | | | | | | | |
| Participants in category, N | 47,892 | 3,377 | 33,115 | 4,978 | 5,046 | 944 | 432 |
| ^a^HR (95% CI),  P-value |  | 0.52 (0.21-1.27)  0.149 | Reference | 1.65 (1.05-2.58)  0.029 | 2.78 (1.94-4.00)  <0.001 | 3.99 (2.14-7.46)  <0.001 | 9.96 (5.44-18.22)  <0.001 |
| ^b^aHR (95% CI)  P-value |  | 0.52 (0.21-1.27)  0.152 |  | 1.64 (1.05-2.56)  0.030 | 2.79 (1.94-4.00)  <0.001 | 3.89 (2.08-7.27)  <0.001 | 9.73 (5.31-17.80)  <0.001 |
| ^c^aHR (95% CI)  P-value |  | 0.66 (0.27- 1.62)  0.367 |  | 1.15 (0.74-1.79)  0.542 | 1.46 (1.00-2.12)  0.048 | 1.42 (0.73-2.79)  0.303 | 2.58 (1.25-5.34)  0.010 |

Underweight- BMI <5th percentile, normal weight- BMI 5th-84.9th percentile, overweight- BMI 85th-94.9th percentile, obese- BMI ≥95th percentile, not including class 2 and class 3 obesity, class 2 obesity- BMI ≥120% to <140% of the 95^th^ percentile or BMI ≥35 to <40 kg/m^2^, class 3 obesity- BMI ≥140% of the 95^th^ percentile or BMI ≥40 kg/m^2^. SD-standard deviation, 95% CI- 95% of the confidence interval, HR- Hazard ratio, aHR- adjusted Hazard ratio

^a^Unadjusted, ^b^Adjusted to socio-economic factors, ^c^ Adjusted to socio-economic factors and adult BMI
