# Supplementary material for: Late-adolescent weight categories and early kidney disease in young adulthood: a nationwide study of Arab and Jewish Israelis
Source: Pediatr Nephrol. 2026 Feb 23;41(7):2131–43. doi: 10.1007/s00467-026-07197-7 (PMC13197338; doi:10.1007/s00467-026-07197-7)
Supplement: Supplementary file 7 — Supplementary file7 (DOCX 26 KB) [file 467_2026_7197_MOESM7_ESM.docx]

**Article title:** Ethnic differences in the association of weight categories in adolescence with early kidney disease in young adulthood - a nationwide study

**Journal name:** Pediatric Nephrology

**Author names:** Yulia Treister-Goltzman

**Affiliation and e-mail address of the corresponding author:** Yulia Treister-Goltzman, [yuliatr@walla.com](mailto:yuliatr@walla.com)

**Online Resource 6.** Association between adolescent weight category and incident early kidney disease in young adulthood using inverse probability weighting for missing microalbumin values

|  | **Total** | **Weight categories in adolescence** | | | | | |
| --- | --- | --- | --- | --- | --- | --- | --- |
|  |  | **Underweight** | **Normal** | **Overweight** | **Obese** | **Class 2 obesity** | **Class 3 obesity** |
| ***Arab ethnicity*** | | | | | | | |
| Participants in category, N | 53,492 | 1,900 | 39,083 | 6,276 | 5,096 | 825 | 312 |
| ^a^HR (95% CI),  P-value |  | 0.51 (0.18-1.40)  0.190 | Reference | 1.15 (0.80-1.65)  0.450 | 2.28 (1.73-2.99)  <0.001 | 3.32 (2.14-5.15)  <0.001 | 5.80 (3.51-9.59)  <0.001 |
| ^b^aHR (95% CI)  P-value |  | 0.51 (0.19-1.41)  0.196 |  | 1.15 (0.80-1.65)  0.465 | 2.28 (1.73-3.00)  <0.001 | 3.34 (2.15-5.19)  <0.001 | 5.79 (3.50-9.57)  <0.001 |
| ^c^aHR (95% CI)  P-value |  | 0.65 (0.24- 1.78)  0.404 |  | 0.89 (0.61-1.28)  0.524 | 1.42 (1.06-1.91)  0.019 | 1.59 (0.99-2.56)  0.058 | 2.18 (1.26-3.79)  0.005 |
| ***Jewish ethnicity*** | | | | | | | |
| Participants in category, N | 47,892 | 3,377 | 33,115 | 4,978 | 5,046 | 944 | 432 |
| ^a^HR (95% CI),  P-value |  | 0.57 (0.23-1.40)  0.220 | Reference | 1.23 (0.79-1.94)  0.359 | 1.60 (1.11-2.30)  0.012 | 1.64 (0.88-3.07)  0.122 | 3.63 (1.97-6.68)  <0.001 |
| ^b^aHR (95% CI)  P-value |  | 0.57 (0.23-1.27)  0.215 |  | 1.23 (0.79-1.93)  0.358 | 1.59 (1.10-2.29)  0.013 | 1.62 (0.87-3.04)  0.129 | 3.62 (1.97-6.65)  <0.001 |
| ^c^aHR (95% CI)  P-value |  | 0.66 (0.27- 1.64)  0.376 |  | 0.98 (0.62-1.53)  0.919 | 1.04 (0.70-1.55)  0.830 | 0.86 (0.43-1.71)  0.660 | 1.54 (0.73-3.29)  0.259 |

Underweight- BMI <5th percentile, normal weight- BMI 5th-84.9th percentile, overweight- BMI 85th-94.9th percentile, obese- BMI ≥95th percentile, not including class 2 and class 3 obesity, class 2 obesity- BMI ≥120% to <140% of the 95^th^ percentile or BMI ≥35 to <40 kg/m^2^, class 3 obesity- BMI ≥140% of the 95^th^ percentile or BMI ≥40 kg/m^2^. SD-standard deviation, 95% CI- 95% of the confidence interval, HR- Hazard ratio, aHR- adjusted Hazard ratio

^a^Unadjusted, ^b^Adjusted to socio-economic factors, ^c^ Adjusted to socio-economic factors and adult BMI
